# Antimicrobial activity of NO-releasing compounds against periodontal pathogens

**DOI:** 10.1371/journal.pone.0199998

**Published:** 2018-10-04

**Authors:** Ji Suk Shim, Dong-sik Park, Dong-Heon Baek, Nayansi Jha, Serk In Park, Hyoung Jin Yun, Won Jong Kim, Jae Jun Ryu

**Affiliations:** 1 Department of Dentistry, Korea University Ansan Hospital, Ansan-si, Republic of Korea; 2 Department of Chemistry, Pohang University of Science and Technology (POSTECH), Pohang, Republic of Korea; 3 Department of Oral Microbiology and immunology, College of Dentistry, Dankook University, Cheonan, Republic of Korea; 4 Korea University Graduate School, Seoul, Republic of Korea; 5 Department of Biochemistry and Molecular Biology, Korea University College of Medicine, Seoul, Republic of Korea; 6 Department of Dentistry, Korea University Anam Hospital, Seoul, Republic of Korea; New York Medical College, UNITED STATES

## Abstract

This study describes the successful synthesis of nitric oxide (NO)-releasing compounds with biodegradable and injectable properties and demonstrates that the kinetics of NO release vary according to the type of NO donor. The antimicrobial activity of NO-releasing compounds against three common periodontal pathogens, i.e., *Aggregatibacter actinomycetemcomitans*, *Porphyromonas gingivalis*, and *Actinomyces israelii*, was investigated using a susceptibility assay. Human gingival fibroblasts were treated with NO-releasing compounds at the minimum concentrations required for bacterial growth and cytotoxicity was evaluated using the MTT cell proliferation assay. Our results suggest that NO-releasing compounds can be used topically to treat both gram-negative and gram-positive periodontal pathogens. Comparison of the antimicrobial activity and cytotoxicity assay results between the NO-releasing compounds revealed that an NO donor comprising a macromolecule without surface charge, a lower instantaneous NO concentration, and an adequate supply of NO were associated with a strong bactericidal effect and low cytotoxicity. NO-releasing compounds with these properties may be suitable for treatment of periodontitis.

## Introduction

Antibacterial agents can be administered systemically or topically for control of infection in periodontal tissue. Systemic administration of antibiotics has been proven to be effective in suppressing progression of periodontitis [1]. However, overuse of antibiotics can have systemic side effects and promote development of antibiotic-resistant pathogens, so periodontitis cannot be treated via the systemic route for extended periods [2]. Antibiotics delivered topically can reach the base of the periodontal pockets, which act as a natural drug reservoir and allow antibacterial concentrations to be maintained for long enough to have an antimicrobial effect [3].

Biodegradable and injectable materials are suitable for topical delivery of antibiotics. Non-biodegradable materials should be removed after the antibiotic is released and may cause irritation and inflammation at the treated site. A biodegradable vehicle has the advantage of keeping patient visits to a minimum. Injection by syringe is a simple procedure for delivery of antibiotic agents and ensures compliance. The procedure requires standard and inexpensive devices, and the antibiotic can be applied rapidly and easily without pain or discomfort to the patient [4].

Nitric oxide (NO), a gaseous reactive free radical species produced in mammalian cells, plays a crucial role in the immune response to pathogens [5] and is implicated in the angiogenesis that occurs at sites of inflammation. NO and reactive oxygen intermediates, such as NO radicals, nitrogen dioxide, dinitrogen trioxide, and peroxynitrite, have bactericidal effects via deamination of DNA, strand breakage, and peroxidation of lipids [6] and could potentially be used as antimicrobial agents. In view of its broad-spectrum antibacterial activity and efficient targeting of both free-floating bacteria and bacterial biofilms, exogenous NO holds promise as an antibacterial agent in various medical fields.

Several types of NO donors, i.e., functional moieties or molecules that generate NO, have been developed for exogenous delivery of NO [7]. Diazeniumdiolates containing the [N(O)NO]^-^ functional group are able to generate two molecules of NO from each moiety, so have been widely used to construct NO delivery systems. These NO donors can be easily functionalized onto any material containing secondary amine groups. NO-releasing compounds containing diazeniumdiolates have now been synthesized using various scaffold molecules, and their antimicrobial activity and cytotoxicity have been investigated. Interestingly, it was found that a macromolecular-based NO delivery system had greater antibacterial activity than a delivery system containing a small-molecule NO donor [8]. The antibacterial activity of NO-releasing compounds against free-floating periodontal pathogens [9] and dental biofilm [10] has been demonstrated. These compounds have also been confirmed to be safe when used in the oral cavity [11]. In a previous study, we investigated the cytotoxic effects of NO-releasing compounds synthesized by addition of diazeniumdiolate to a water-soluble Pluronic-branched polyethyleneimine conjugate [12]. Pluronic copolymer is approved by the US Food and Drug Administration (FDA) for use as a support scaffold and has no apparent cytotoxicity, so has good biocompatibility. This tri-block copolymer is also a good substrate for injectable hydrogel and is biodegradable [13].

In this study, we investigated the bactericidal effect of Pluronic F68-branched polyethylenimine-NONOates (F68-BPEI-NO) on three periodontal pathogens, i.e., *Aggregatibacter actinomycetemcomitans*, *Porphyromonas gingivalis*, and *Actinomyces israelii*. Two small-molecule NO donors, i.e., pyrrolidine-NONOates (Py-NO) and diethyltriamine-NONOates (DETA-NO), which release NO at different rates, were used to evaluate the effect of different NO delivery systems. The cytotoxicity of the NO-releasing system was evaluated in human gingival fibroblasts (HGF-1) to assess the possibility of its therapeutic use in oral disease.

## Materials and methods

### Synthesis of NO-releasing compounds

A Pluronic F68-branched polyethyleneimine conjugate (F68-BPEI), the precursor of the polymer with an additional NONOate species (F68-BPEI-NONOates), was synthesized according to a previously described method with some modifications [14]. First, 1.79 g of 4-nitrophenyl chloroformate (pNPC), dissolved in 40 ml of dichloromethane (DCM), was added dropwise to 10 g of Pluronic F68 containing 50 ml of DCM to activate the Pluronic F68. After stirring overnight, the pNPC-conjugated Pluronic F68 (pNPC-F68) was purified by precipitation with cold diethyl ether. Next, 2.84 g of F68-pNPC was dissolved in 23.2 ml of DCM to generate a conjugating branched polyethyleneimine (BPEI). Thereafter, 1.76 g of BPEI 1.8 k was dissolved in 40 ml of DCM with 6 ml of TEA and the mixture was stirred at 0°C for 15 min. After stirring, the F68-pNPC solution was added dropwise to the BPEI-containing solution. The resulting solution was stirred overnight and then warmed to room temperature. After the reaction, the product was purified by precipitation with cold diethyl ether. The remaining BPEI was removed by dialysis, after which the final product was freeze-dried for storage.

Py-NO, DETA-NO, and F68-BPEI-NO were synthesized from each precursor (pyrrolidine, diethylenetriamine, and F68-BPEI, respectively) by addition of NONOates to the secondary amine moieties. Pyrrolidine (1.64 ml) was mixed with acetonitrile (5 ml), ether (5 ml), and 30% sodium methoxide (NaOMe) solution (3.9 ml). Diethylenetriamine (0.52 ml) was mixed with acetonitrile (15 ml) and NaOMe solution (0.9 ml). F68-BPEI dissolved in 20 ml of methanol (MeOH) was mixed with 3 ml of tetrahydrofuran and 0.2 ml of 30% NaOMe solution. Each precursor-containing solution was placed in a high-pressure NO reactor with argon purging at several intervals. The reactor was immediately filled with NO gas at 90 psi and maintained at this pressure for 3 days to functionalize the diazeniumdiolates. After flushing with argon gas at 20 psi, Py-NO and DETA-NO were collected by filtration and washed in cold diethyl ether. F68-BPEI-NO was obtained by precipitation with cold diethyl ether. After collection of the products, each product was placed under vacuum to remove any remaining solvent, and then sealed and stored at −20°C.

### Measurement of NO release

The amount of NO released was measured using a Sievers 280i Nitric Oxide Analyzer (GE Healthcare, Boulder, CO, USA), which measures chemiluminescence. The NONOate-containing materials were added to Dulbecco’s phosphate-buffered saline, and the release profile was obtained. The DETA-NO release profile was analyzed up to its detection limit (1 pmol). NO release was evaluated by characterizing multiple NO parameters, including [NO]_t_ (number of moles of NO released per mg of the material), t_d_ (duration of NO above 1 pmol/s), [NO]_m_ (maximum instantaneous concentration of NO released), t_m_ (time required to reach [NO]_m_), and t_1/2_ (half-life of NO released). For the bactericidal and cytotoxicity assays, the NO compounds were brought into contact with bacteria and cells, respectively, 10 min after they were mixed with solution. The kinetics of NO release were recorded at 0 and 10 min after the initial NO release response.

### Bacterial strains and cultivation

*Aggregatibacter actinomycetemcomitans* (ATCC 43718), *Porphyromonas gingivalis* (ATCC 33277), and *Actinomyces israelii* (ATCC 12102) were purchased from the American Type Culture Collection (ATCC, Manassas, VA, USA). For verification of decontamination, *P*. *gingivalis* was cultured in trypticase soy agar supplemented with 5% sheep blood, hemin (1 μg/ml), and vitamin K (0.2 μg/ml), and *A*. *actinomycetemcomitans* and *A*. *israelii* were grown in brain heart infusion (BHI) agar at 37°C under anaerobic conditions (5% H_2_, 10% CO_2_, and 85% N_2_). A single colony of each type of bacterium was then cultivated with the appropriate medium. For the susceptibility assay, *P*. *gingivalis* was cultivated in BHI broth containing hemin (1 μg/ml) and vitamin K (0.2 μg/ml) and *A*. *actinomycetemcomitans* and *A*. *israelii* were cultivated in BHI broth at 37°C under anaerobic conditions. The bacteria were then counted using a bacteria counting chamber (Marienfeld, Lauda-Konigshofen, Germany) and the concentration of each type of bacteria was adjusted to 1 × 10^7^ cells/ml by addition of fresh medium.

### Antimicrobial assay of NO compounds

The antimicrobial activity of the NO-releasing compounds against periodontal pathogens was evaluated using a susceptibility assay according to the methods of the Clinical and Laboratory Standards Institute [15]. A minimum inhibitory concentration (MIC) assay was carried out using a 96-well polystyrene plate (SPL Life Sciences, Gyeonggi, Korea). Next, 180-μl aliquots of BHI broth containing hemin and vitamin K for *P*. *gingivalis* or BHI broth for *A*. *actinomycetemcomitans* and *A*. *israelii* were dispensed into the wells of the plate. A solution containing Py-NO, DETA-NO, or FBN dissolved in sterilized, distilled H_2_O was added to the 12th column of the 96-well plate and serially diluted 2-fold progressing to the second column using a multi-channel micropipette. The first column of each well contained broth only and served as a negative control. Finally, 20-μl bacterial suspensions (containing 1 × 10^5^ cells) were inoculated into each well. The plates were then incubated at 37°C for 36 h under anaerobic conditions. Bacterial growth was measured by optical density at a wavelength of 660 nm using a microplate reader (SpectraMax M2, Molecular Devices, Sunnyvale, CA, USA). A 96-well plate containing medium with dilution of NO-releasing compounds and inoculation of periodontal pathogens was incubated for evaluation of the minimum bactericidal concentration (MBC). A 50-μl aliquot of liquid from each well was inoculated and spread onto BHI agar for *A*. *actinomycetemcomitans* and *A*. *israelii* or onto trypticase soy agar containing sheep blood, hemin, and vitamin K for *P*. *gingivalis*. The plates were then incubated at 37°C for 3 days (*A*. *actinomycetemcomitans* and *A*. *israelii*) or 5 days (*P*. *gingivalis)* under anaerobic atmospheric conditions. The lowest concentration that revealed no visible bacterial growth after subculturing was taken to be the MBC.

### In vitro cytotoxicity

HGF-1 (ATCC #CRL-2014) cells were cultured in Dulbecco’s modified Eagle’s medium supplemented with 10% fetal bovine serum and 1% penicillin/streptomycin and incubated at 37°C under humidified conditions in 5% CO_2_. The cells were seeded with medium (100 μl) in a 96-well plate (SPL Life Sciences) at a density of 1 × 10^4^ cells/well and incubated at 37°C for 24 h. Solutions (100 μl) of control medium and NO-releasing medium prepared in Dulbecco’s modified Eagle’s medium by the same method as that used for the bactericidal assay were added to the cells and incubated at 37°C for 24 h. Next, 20 μl of a 5 mg/ml solution of MTT was added to each well and incubated for 4 h in the dark; 100 μl of medium was then aspirated carefully from each well and 100 μl of dimethyl sulfoxide was added to each well to dissolve the formazan precipitate. Absorption was measured at a wavelength of 570 nm using a VERSAmax tunable microplate reader (Molecular Devices). The viability of the cells in each well was calculated by comparing it with that of an untreated control (100%).

### Statistical analysis

Differences between cell and bacterial samples treated or not treated with the NO-releasing compounds were assessed for statistical significance using the Kruskal-Wallis test and the Mann-Whitney *U* test, using SPSS version 23 software (IBM Corp., Armonk, NY, USA). A p-value <0.05 was considered statistically significant.

## Results

### Kinetics of NO release

**Fig 1:**
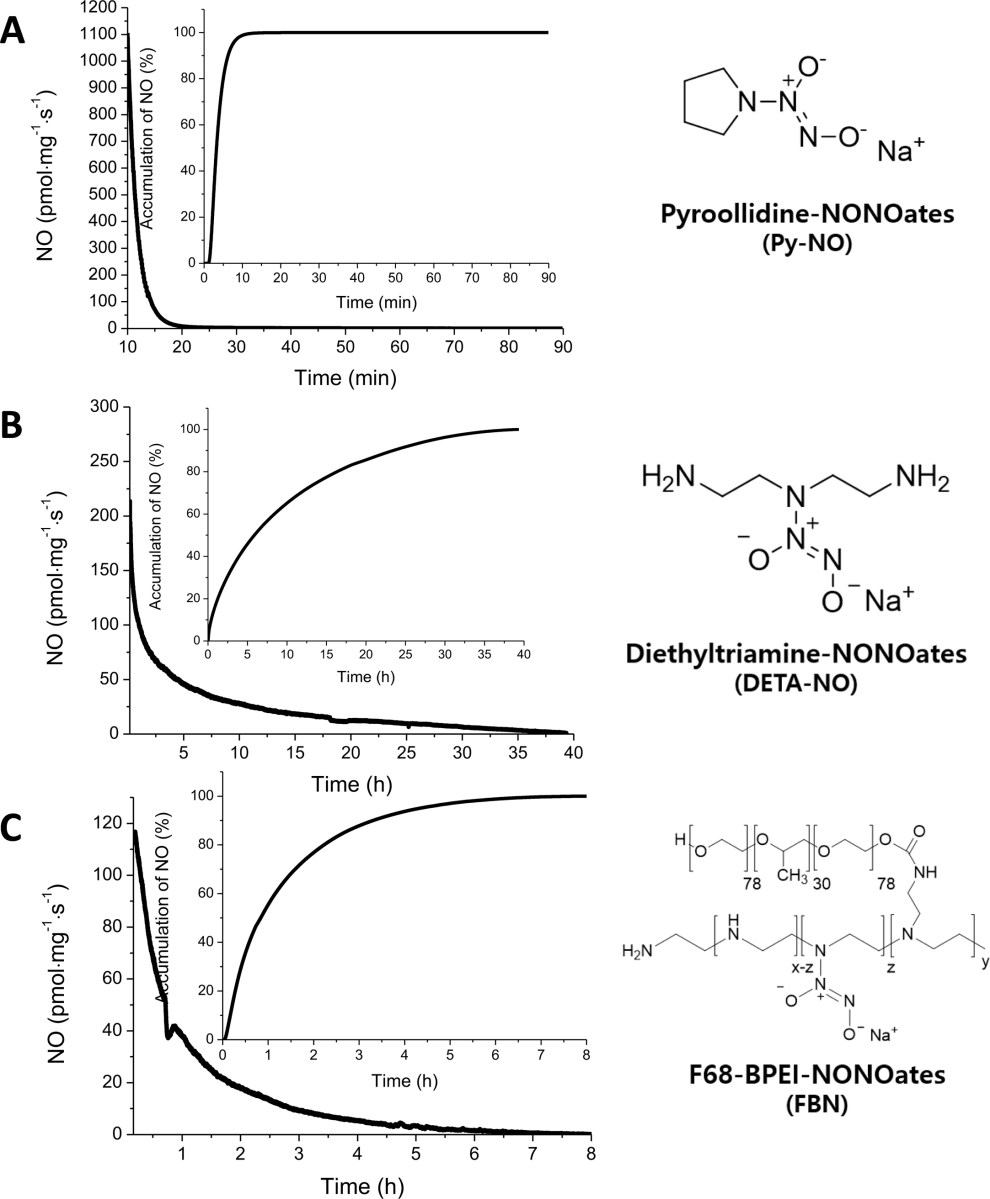
Characterization of NO-releasing compounds. “Time” indicates the time course after the bacteria or cells were exposed to NO. NO, nitric oxide.

Release of NO from Py-NO, DETA-NO, and F68-BPEI-NO was characterized using chemiluminescence in Dulbecco’s phosphate-buffered saline at 37°C. The number of moles of NO released per mg, duration of NO release, maximum instantaneous concentration of NO released, time required to reach [NO]_m_, time required to reach the half-life of NO release, number of moles of NO released per mg from 10 min to 24 h, and the instantaneous concentration of NO released at 10 min were recorded for each compound (Table 1). Fig 1 shows the time course of NO release and the cumulative NO release profiles after 10 min. Py-NO showed rapid NO release with a high instantaneous NO concentration (49,227 pmol/mg/sec at 0 min, 1105.5 pmol/mg/sec at 10 min) for a short duration (1.5 h). In contrast, DETA-NO showed a slow NO release with a relatively lower instantaneous concentration (546.3 pmol/mg/sec at 0 min, 213.8 pmol/mg/sec at 10 min) for a long duration (39.3 h). F68-BPEI-NONOates showed an intermittent NO release speed with a much lower instantaneous NO concentration (114.7 pmol/mg/sec at 0 min, 115.1 pmol/mg/sec at 10 min) and NO release was complete after 8.06 h. The calculated NO capacity from 1 mole of each molecule was evaluated from the NO release profile and the molecular weight of each material (Py-NO, 1.48 mole NO/mol; DETA-NO, 0.609 mole NO/mol; F68-BPEI-NO, 4.87 mole NO/1 mol).

**Table 1:** NO release properties of tested materials^a^.

	[NO]_t_^b^	t_d_^c^	[NO]_m_^d^	t_m_^e^	t_1/2_^f^	[NO]_t,10p_^g^	[NO]_i,10p_^h^
**Py-NO**	9673	1.50	49227	2.2	3.25	119.9	1105.5
**DETA-NO**	3290	39.3	546.3	3.0	354	2802	213.8
**F68-BPEI-NO**	437	8.06	114.7	12.5	50.1	393.0	115.1

^a^Measured in Dulbecco’s phosphate-buffered saline at 37°C using a NOA 280i chemiluminescence NO analyzer.

^b^[NO]_t_, total number of nanomoles of NO released per mg (nmol/mg).

^c^t_d_, duration of NO release (h).

^d^[NO]_m_, maximum instantaneous concentration of NO released (pmol/mg/sec).

^e^t_m_, time required to reach [NO]_m_ (min).

^f^t_1/2_, half-life of NO release (min).

^g^[NO]_t,10p_, number of nanomoles of NO released per mg from 10 min to 24 h (nmol/mg).

^h^[NO]_i,10p_, instantaneous concentration of NO release (pmol/mg/sec). NO, nitric oxide

### Antimicrobial activity of NO-releasing compounds against periodontal pathogens

**Fig 2:**
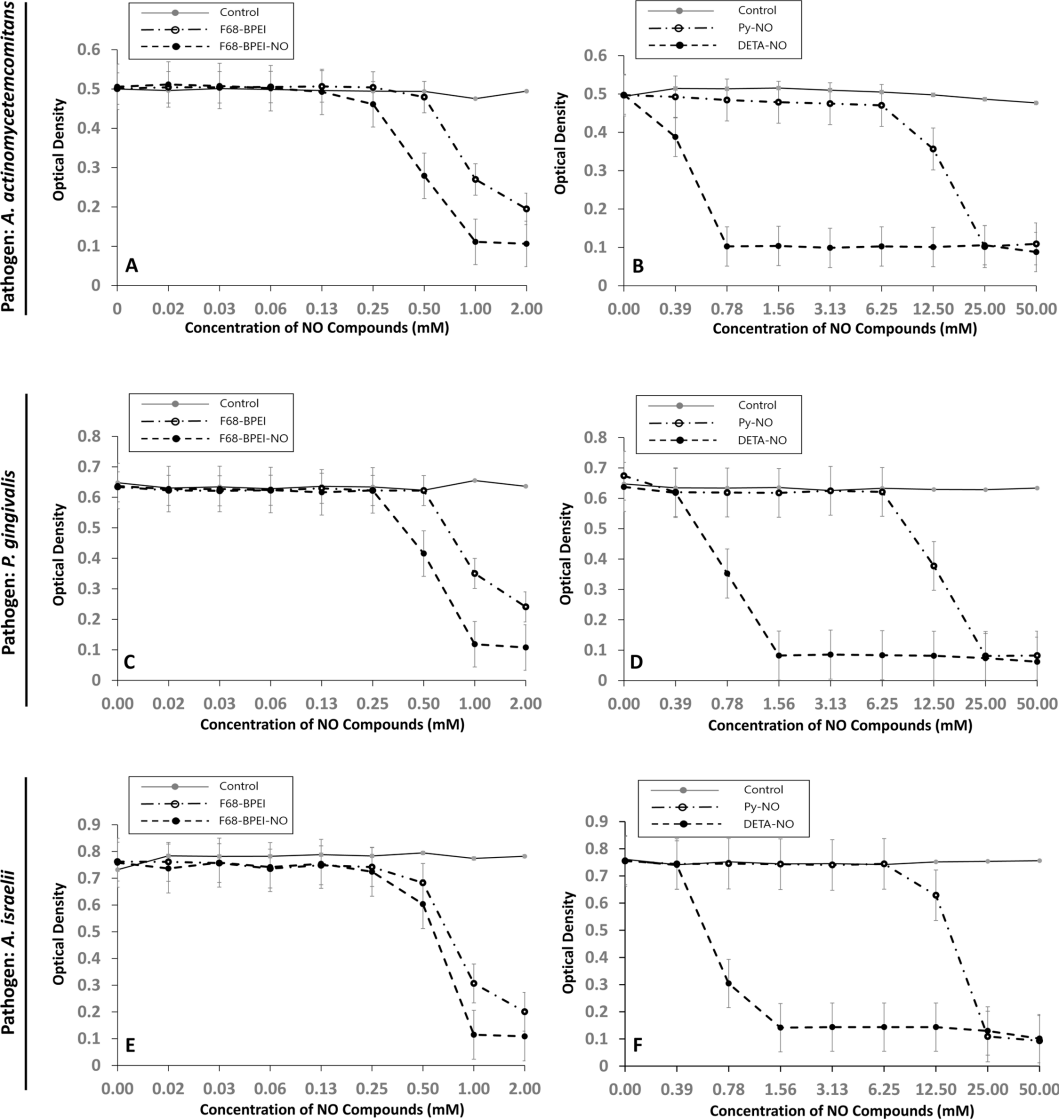
Bactericidal efficacy of NO-releasing compounds.

F68-BPEI-NO, DETA-NO, F68-BPEI, and Py-NO inhibited growth of *P*. *gingivalis* at concentrations at or above 0.5 mM, 0.78 mM, 1 mM, and 12.5 mM, respectively. Lower MICs were observed for each compound against *A*. *israelii* (0.5 mM, 0.78 mM, 1 mM, and 25 mM, for F68-BPEI-NO, DETA-NO, F68-BPEI, and Py-NO, respectively). To inhibit the growth of *A*. *actinomycetemcomitans*, the concentrations of NO-releasing compounds required were 0.39 mM for DETA-NO, 1.5 mM for F68-BPEI-NO, 1 mM for F68-BPEI, and 12.5 mM for Py-NO (Fig 2). F68-BPEI-NO achieved greater inhibition of bacterial growth than its precursor, F68-BPEI.

The MBC was also measured to analyze the bactericidal effect of the NO-releasing materials in more detail. The MBC for F68-BPEI-NO, DETA-NO, and Py-NO was observed at 1 mM, 1.6 mM, and 25 mM, respectively, against *P*. *gingivalis* and 1 mM, 1.6 mM, and 25 mM against *A*. *israelii*. *Actinomycetemcomitans* was more sensitive to DETA-NO, with the MBC of DETA-NO, F68-BPEI-NO, and Py-NO being observed at 0.78 mM, 1 mM, and 25 mM, respectively.

The bactericidal effects of Py and DETA were also investigated to confirm that the effects were attributable to release of NO; both compounds inhibited the growth of periodontal pathogens at very high concentrations. Unlike Py-NO and DETA-NO, Py and DETA showed antimicrobial activity at a concentration of 10-fold or more (S1 Fig).

### Cytotoxicity to HGF-1 cells

**Fig 3:**
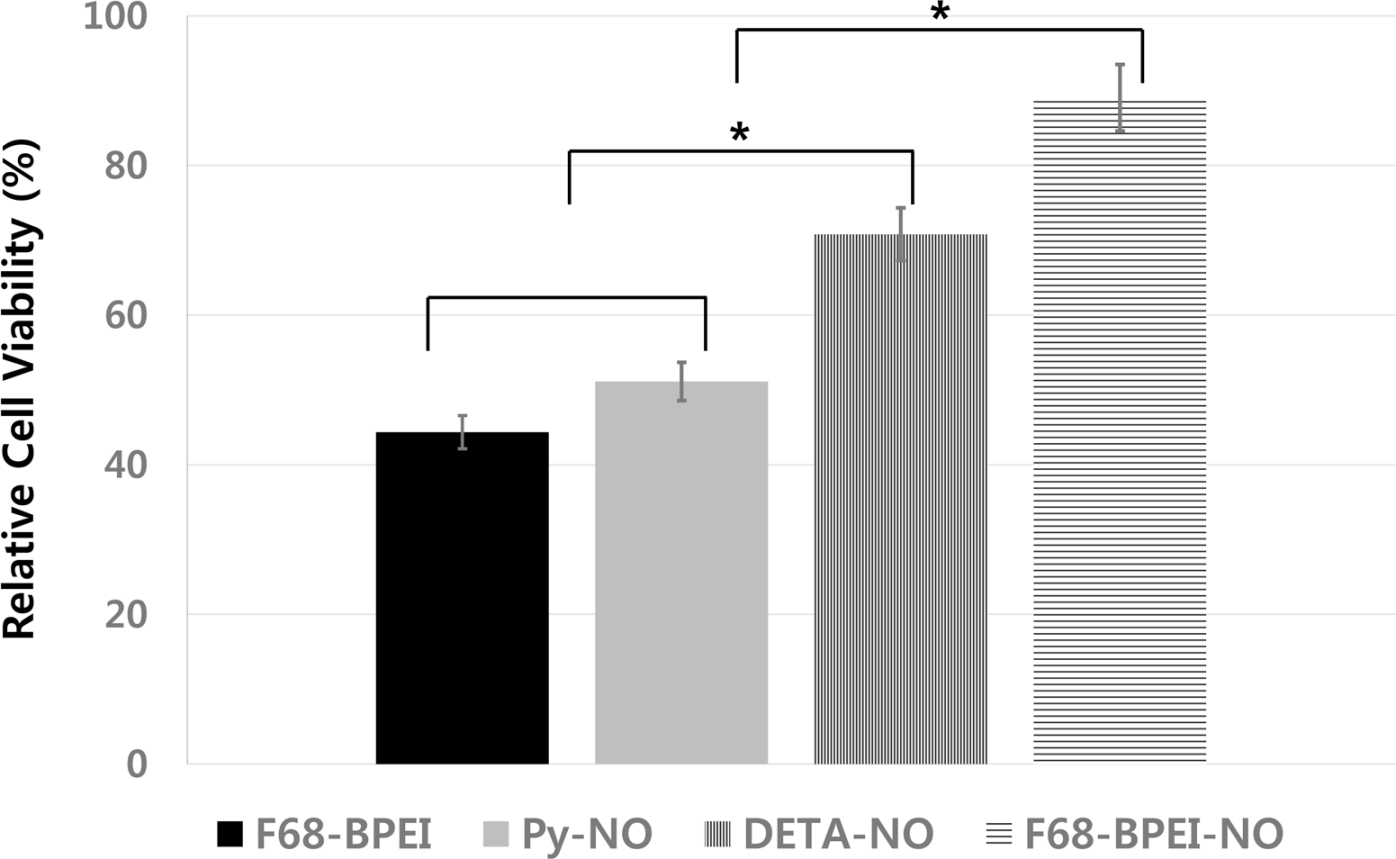
Cytotoxic effects of NO-releasing compounds on HGF-1 cells. The bactericidal assay showed that the minimum concentrations of NO-releasing compounds required to kill all pathogens within 24 h were 2, 1, 25, and 1.56 mM for F68-BPEI, F68-BPEI-NO, Py-NO, and DETA-NO, respectively. The cell viability in each group was calculated by comparing the viability with that of an untreated control (100%). Asterisks indicate significant differences (*P* <0.05) between groups.

The cytotoxicity of the NO-releasing compounds was investigated in HGF-1 cells by determination of the difference in metabolic activity between cells treated or not with the compounds (Fig 3). When effective killing concentrations of the NO-releasing compounds were used (2 mM, 1 mM, 25 mM, and 1.56 mM for F68-BPEI, F68-BPEI-NO, Py-NO, and DETA-NO, respectively), their cytotoxicity values were 44.35%, 89.06%, 51.13%, and 70.80%, respectively.

## Discussion

This study examined the potential suitability of NO-releasing compounds for use in clinical practice as antibacterial agents against periodontal pathogens. Py and DETA are organic compounds with monomolecular structures that are soluble to water and polar organic solvents [16,17]. Although F68-BPEI is an organic compound with a macromolecular structure, it is water-soluble and is biodegradable [18]. Moreover, when mixed with a solvent such as phosphate-buffered saline or distilled water, the physical state of F68-BPEI varies from a liquid at low temperatures to a gel at body temperature [18]. Therefore, F68-BPEI may be a suitable substrate for an injectable hydrogel and applied directly to treat patients with periodontitis [14]. In contrast, Py and DETA can be used in an injectable form after being mixed with polyethylene glycol ([19]. To evaluate the bactericidal efficiency of NO, bactericidal assays were performed against *A*. *actinomycetemcomitans* (gram-negative), *P*. *gingivalis* (gram-negative), and *A*. *israelii* (gram-positive). The cytotoxic effects of the NO-releasing compounds on HGF-1 cells at the MBC were measured to evaluate the therapeutic potential of these agents.

Like in previous studies, the kinetics of NO release varied depending on the NO delivery system used [20,21], although all NO-releasing compounds in this study used the same source of NO (NONOates). Py and DETA are mono-molecules, but the NO-releasing compounds derived from them release NO differently. Although Py-NO has a higher NO capacity compared with the other compounds, the NO release kinetics are much more rapid than those of other similar structures [22]. Therefore, Py-NO may exhibit low antimicrobial activity against periodontal pathogens. In the case of DETA-NO, the remaining amine group would be positively charged because of protonation in aqueous conditions, and the compound would transition to a zwitterion form; this form stabilized the NONOates group, resulting in prolonged release of NO [23]. F68-BPEI is a macromolecule that showed the lowest instantaneous NO concentration value, with an intermediate duration of release and total amount of NO. The reason for the longer duration of NO release from F68-BPEI than from Py-NO is similar to that for DETA-NO.

*A*. *actinomycetemcomitans* and *P*. *gingivalis* are gram-negative periodontal pathogens that were susceptible to NO. This finding corresponds to the results of previous studies [9,20]. Py-NO, which released a higher instantaneous NO concentration with a lower amount of NO, showed higher MICs and MBCs than DETA-NO, which released a lower instantaneous NO concentration with a larger amount of NO. These results confirm that the instantaneous NO concentration was less critical for killing periodontal pathogens than total intracellular NO delivered over time [9,20]. Furthermore, it has been demonstrated that continuous exposure of bacteria to gaseous NO is required for an antibacterial effect [24]. Therefore, the duration of NO release from each compound is a major determinant of antibacterial efficacy.

Bactericidal efficacy depends on surface charge as well as the kinetics of NO release; bacterial cell walls are negatively charged, and positively charged macromolecules can interact with and disrupt bacterial cell membranes via electrostatic interaction [25]. F68-BPEI had an inhibitory effect on periodontal pathogens without NO that may be attributable to the high positive charge of F68-BPEI itself (20 mV). Compared with Py-NO and DETA-NO, F68-BPEI-NO had a bactericidal effect with less NO. The factor underlying these results seems to be the total molecular mass of the NO donor. Previous studies have shown that donors composed of macromolecules have greater NO activity against bacteria than donors composed of mono-molecules [22,26] because of the localization of NO on the macromolecules [22].

We evaluated the bactericidal efficiency of NO against *A*. *israelii*, a gram-positive periodontal pathogen and found results similar to those of the bactericidal assays against gram-negative periodontal pathogens. *A*. *israelii* is only one of the strains of bacteria that cause gingivitis; *Actinomyces* spp. are also significant pathogens in the etiology of medication-related osteonecrosis of the jaw (MRONJ) [27,28]. Therefore, the results of this study suggest that NO should be considered for the treatment of MRONJ. NO may have additional benefits in the treatment of MRONJ because it promotes angiogenesis [29] and an anti-inflammatory response [30], and also plays a role in attenuating the adverse effects of bisphosphonates in human gingival fibroblasts [12].

F68-BPEI becomes strongly positive when secondary amines are attached, causing a non-specific ionic interaction with negatively charged bacterial membranes and zwitterionic cell membranes. Therefore, F68-BPEI is highly cytotoxic and also inhibits growth of periodontal pathogens. Interestingly, in the present study, the cytotoxic effect of F68-BPEI-NO was improved after addition of NONOates. The positive charge of F68-BPEI (20.88 mV) was reduced when attached to NONOates, and F68-BPEI-NO had a surface charge that was close to zero (0.43 mV). NO was added at a relatively low concentration, suggesting that inducing zero surface charge by addition of NO may have a positive effect on cells. Although a positive surface charge can improve the bactericidal effect, a lack of surface charge seems to be more beneficial for the therapeutic use of NO with respect to cytotoxicity. Py-NO was more cytotoxic than DETA-NO in our study, which confirms that an instantaneous NO concentration is more important for increasing cytotoxicity than the total amount of NO released [9,31]. Therefore, a lower instantaneous NO concentration with a greater total amount of NO appears to have a better bactericidal effect and be less cytotoxic, and a macromolecule may be favorable as a NO delivery system because of the high efficiency of NO against pathogens.

Eradication of microorganisms from the periodontal pocket is the most important step in treating periodontitis. The limited effects of mouth rinsing and irrigation have prompted research on alternative antimicrobial delivery systems. Advances in drug delivery technology have allowed development of controlled-release drug formulations. The requirements for a drug delivery system to treat periodontitis include maintenance of an effective drug concentration for an adequate duration at the site of infection and minimal or no side effects in periodontal tissue [32]. Various devices, including fibers [33], films [34], strips [33,35], gels [36], and microparticles [37], have been developed for this purpose, and most of the commercially available injectable and biodegradable carriers have the advantages of being cost-effective, convenient to use, and less painful when administered to patients [32]. The antibiotics used in these carriers include metronidazole, minocycline, chlorhexidine, and tetracycline; however, patients with chronic periodontitis often have subgingival periodontal pathogens that are resistant to the antibiotics commonly used in clinical periodontal practice [38]. In the present study, F68-BPEI-NO had an adequate antimicrobial effect against periodontal pathogens and was minimally cytotoxic to gingival fibroblasts, and can be used in an injectable and biodegradable carrier form. Importantly, F68-BPEI-NO could be an effective alternative treatment for chronic periodontitis caused by pathogens resistant to conventional antibiotics, given our previous finding that F68-BPEI-NO had a marked bactericidal effect on methicillin-resistant *Staphylococcus aureus* [39]. However, release of NO is affected by temperature, which differs between experimental conditions and the oral cavity. Research in animals is now needed to confirm the efficacy of NO-releasing compounds in the treatment of periodontal disease. Additional animal studies are also needed to determine how the amounts of NO-releasing compounds delivered to the periodontal pockets change over time. The NO-releasing compounds used in this study do not need modification for use in animal studies because they are biocompatible and injectable. The results of the present study can be used to determine the concentration of NO-releasing compounds needed in such animal studies.

## Conclusion

F68-BPEI-NO may be a prepotent candidate for a topical agent in the treatment of periodontal disease because it demonstrated sufficient bactericidal effects on periodontal pathogens, with minimum cytotoxicity to HGF-1 cells. NO donors composed of a macromolecule without surface charge, a lower instantaneous NO concentration, and sufficient total amount of NO had a strong bactericidal effect and low cytotoxicity.

## Supporting information

S1 Fig Antimicrobial activity of pyrrolidine and DETA against periodontal pathogens. In comparison to py-NO and DETA-NO, pyrrolidine and DETA showed antimicrobial activity at a concentration of 10-fold or more, and their antimicrobial effect was attributable to release of NO.
(TIFF)
