# Supplementary figures and images for: Antimicrobial activity of NO-releasing compounds against periodontal pathogens
Source: PLoS One. 2018 Oct 4;13(10):e0199998. doi: 10.1371/journal.pone.0199998 (PMC6171828; doi:10.1371/journal.pone.0199998)

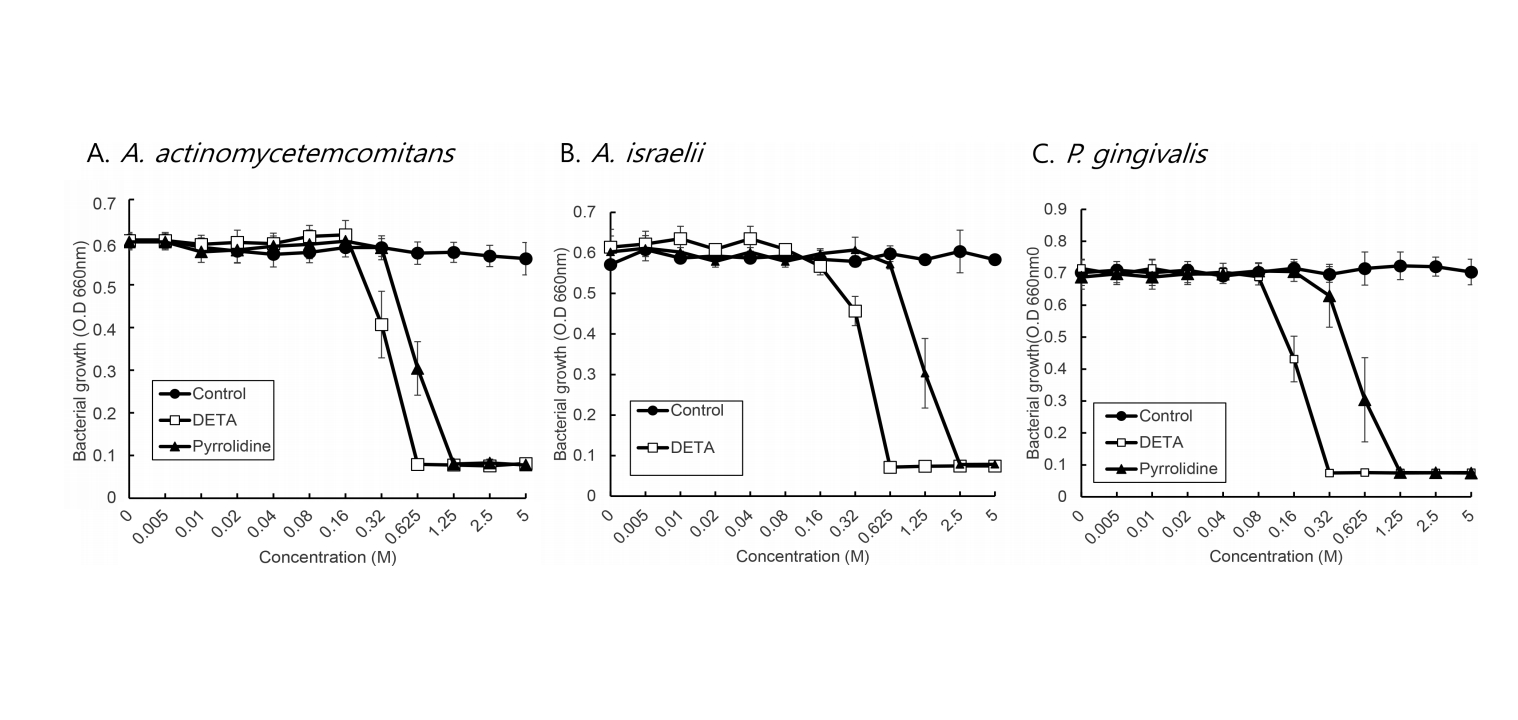

Supplement: S1 Fig — In comparison to py-NO and DETA-NO, pyrrolidine and DETA showed antimicrobial activity at a concentration of 10-fold or more, and their antimicrobial effect was attributable to release of NO. (TIFF) [file pone.0199998.s001.tiff]
